# Supplementary material for: Comparison of Methods for Incorporating Related Data When Developing Clinical Prediction Models: A Simulation Study
Source: Biom J. 2026 Aug 27;68(5):e70166. doi: 10.1002/bimj.70166 (PMC13520826; doi:10.1002/bimj.70166)
Supplement: Supplementary file 2 [file BIMJ-68-e70166-s001.zip › third submission code review - R2/reproducibility_supplement/results/Plots/Individual_plots/cal_plot_logistic_calibration_source_only_model15_predictor_outcome_SC_ID2_TS0.25_DS4.pdf]

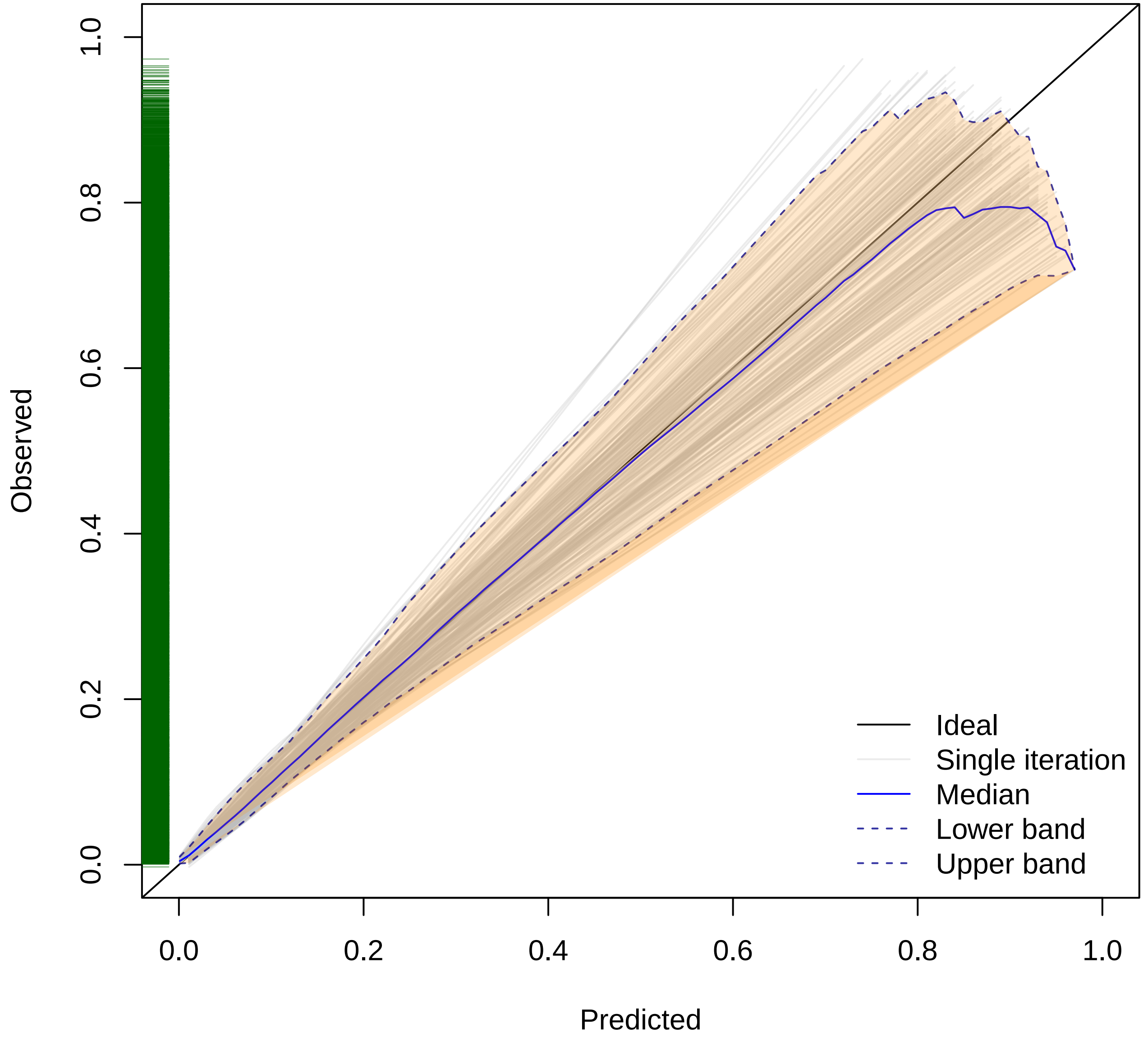

Ideal
Single iteration
Median
Lower band
Upper band
Observed
Predicted
0.0
0.2
0.4
0.6
0.8
1.0
